# Posterior Microphthalmos With RPE Dysfunction Misdiagnosed as Macular Edema

**DOI:** 10.1155/crop/2525345

**Published:** 2025-09-30

**Authors:** Xiaping Wang, Weiwei Du, Shuangnong Li, Hua Fan, Yanjie Li

**Affiliations:** ^1^The First Clinical Medical College, Shanxi Medical University, Taiyuan, China; ^2^Department of Retina, Shanxi Aier Eye Hospital, Taiyuan, China; ^3^Aier Eye Hospital, Jinan University, Guangzhou, China; ^4^Aier Academy of Ophthalmology, Central South University, Changsha, China; ^5^Department of Ophthalmology, The First Clinical Hospital of Shanxi Medical University, Taiyuan, China

**Keywords:** case report, macular edema, posterior microphthalmos

## Abstract

**Background:**

Posterior microphthalmos (PM) typically manifests as retinal thickening, edema, and the presence of papillomacular fold (PMF). We report a case of bilateral PM with macular atrophy and RPE dysfunction. Late-stage of RPE dysfunction in PM is poorly documented in the literature. We present a case that highlights this scenario, aiming to raise awareness of this condition among ophthalmologists.

**Case Presentation:**

The authors emphasize the atypical imaging findings associated with PM and RPE dysfunction. This case was initially misdiagnosed as macular edema, leading to the administration of anti-VEGF treatment, a common clinical pitfall in PM due to intraretinal cystoid changes. Nevertheless, no improvement was observed in retinal thickness, as evidenced by OCT examination, or inpatient visual acuity following anti-VEGF therapy.

**Conclusion:**

Our case adds to recently reported cases linking PM and RPE dysfunction. The recognition of the short axial length and the utilization of imaging modalities such as OCT and FFA contribute to the diagnosis of this condition. It is important to note that anti-VEGF therapy is not applicable for this condition.

## 1. Background

Microphthalmos refers to an ocular condition characterized by an axial length smaller than two standard deviations below the average, typically less than 20 mm. Some studies propose a range between 18 and 20.5 mm [1]. In a more extensive study of the biometric criteria of posterior microphthalmos (PM), axial length ranges were much shorter (15–17 mm on average and almost never exceed 19 mm) [2]. The sclera is abnormally thickened [3]. PM was considered a subtype of microphthalmos.

These structural anomalies contribute to modifications in the choroidal vasculature, elevating the vulnerability to choroidal leakage. Such a condition is frequently associated with various retinal and choroidal changes, including optic disc anomalies, macular schisis, retinal folds, and papillomacular fold (PMF). Meanwhile, microphthalmos are at risk for uveal effusion, exudative retinal detachment, and angle-closure glaucoma [4].

However, for patients with microphthalmos, the clinical manifestations may change with age and disease progression [5]. Structural retinal changes and possible choroidal abnormalities may contribute to secondary Muller cell dysfunction, resulting in characteristic manifestations [6]. There are many reports on the manifestations of nanophthalmos (NO) and PM without retinal pigment epithelium (RPE) changes [7, 8]. However, in these reports, the cases also demonstrated manifestations related to RPE abnormalities. In Zor et al.'s study [7], a case presented features of pigmentary retinitis pigmentosa, and the authors considered it might be associated with gene mutations. In a case series study, the authors used carbonic anhydrase inhibitors (CAIs) to increase fluid transport across the RPE for treating PM-related macular cystoid lesions. One case in this study showed optical coherence tomography (OCT) findings which are similar to our reported case, both demonstrating full-thickness retinal cystoid edema resulting from retinal barrier dysfunction. However, the study did not provide a detailed description of the pathogenesis [9].

We report a case of bilateral PM with macular atrophy and retinal barrier dysfunction with the characteristic multimodal imaging features of the fundus.

## 2. Case Presentation

A 53-year-old male patient, with a bilateral hyperopic history, presented at our ophthalmology clinic with a chief complaint of distorted vision in both eyes over the past 12 months. The patient's uncorrected visual acuity (UCVA) in the right eye is 0.06, and the best corrected visual acuity (BCVA) is 0.15. In the left eye, the UCVA is 0.03, and the BCVA is 0.12. Refraction indicates 1400 diopters (Ds) of hyperopia in the right eye and 1200 Ds of hyperopia in the left eye. His intraocular pressure in both eyes is normal.

His medical history includes a diagnosis of “bilateral macular degeneration” 1 year ago at a hospital. Subsequently, 6 months ago, he received a diagnosis of “bilateral age-related macular degeneration (AMD)” at another hospital and underwent an intravitreal injection (ranibizumab) for the left eye. Despite the intervention, there was no improvement observed in either visual acuity or the distortion of vision. The patient has experienced poor vision in both eyes since childhood, corrected to 0.6–0.7 with glasses. Additionally, he has a 6-year history of hypertension. The patient had previously received topical brinzolamide, but due to suboptimal efficacy, he discontinued the medication on his own.

Comprehensive ocular examinations were conducted for the patient, including IOL-master, fundus fluorescein angiography (FFA), indocyanine green angiography (ICGA), swept-source optical coherence tomography (SS-OCT), optical coherence tomography angiography (OCTA), and spontaneous fundus autofluorescence photography.

**Figure 1:**
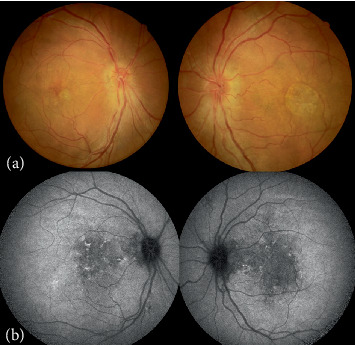
(a) Fundus photography shows macular atrophy and pseudodisc edema in both eyes. (b) Autofluorescence shows fleck-like changes in fluorescence, connected to the optic disc.

**Figure 2:**
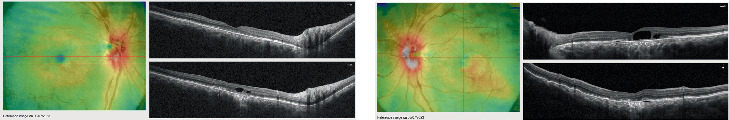
SS-OCT indicated outer retinal atrophy and large cystic changes in the interretinal layers. (a) Right eye and (b) left eye.

**Figure 3:**
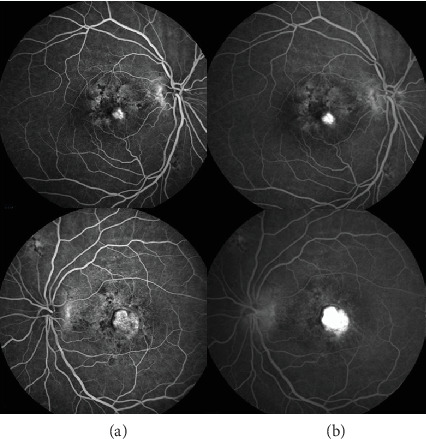
Fluorescein angiography (FFA) shows (a) staining of dye in the early phase and (b) dye pooling in the late stage in the macula of both eyes.

**Figure 4:**
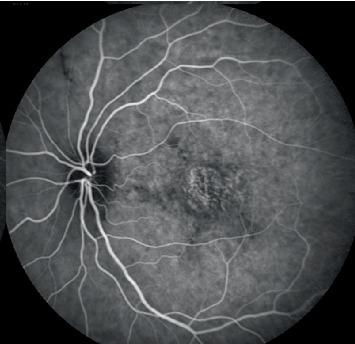
Indocyanine green angiography (ICGA) revealed a “radiating” low-fluorescence pattern at the lesion site.

Examinations showed an axial length of 18.5 mm in the right eye and 18 mm in the left eye; corneal diameter was 11.7 mm in the right eye and 12 mm in the left eye; and keratometry readings were 43.86/45.17 Ds in the right eye and 44.23/45.36 Ds in the left eye. Fundus photography demonstrated pseudodisc edema in both eyes (Figure 1), while SS-OCT indicated outer retinal damage (the ellipsoid zone and RPE layer show disrupted continuity with deposition of hyperreflective punctate substances) and cystic changes in the interretinal layers (from outer nuclear layer to inner nuclear layer) (Figure 2). Early-phase FFA displayed staining in the macular area with late-phase fluorescence pooling (Figure 3), devoid of vascular leakage. Additionally, ICGA revealed a “radiating” low-fluorescence pattern at the lesion site (Figure 4).

## 3. Discussion and Conclusions

PM is a rare congenital anomaly first described by Franceschetti and Gernet [10]. It is characterized by a normal anterior segment and a shortened posterior segment, distinguishing it from NO. The condition is differentiated based on corneal diameter: a diameter of ≥ 11 mm is considered indicative of PM, while a diameter of ≤ 11 mm is associated with NO [11]. The primary clinical features of PM include high hyperopia, PMFs, crowded optic discs, and retinoschisis [12] .

OCT features of PMF in PM have been previously described, including cystoid changes that may be misdiagnosed as cystoid macular edema (CME) and macular fold without involvement of RPE [7]. OCTA has further demonstrated that the nanophthalmic macula is characterized by foveal avascular zone (FAZ) attenuation, capillary tortuosity, foveal folds, and thickened subfoveal choroid [7, 13]. In some earlier reports, FFA of PMF showed no dye leakage [14, 15].

There have also been clinical case reports that PM and PMF-associated cystic changes were misdiagnosed as CME, leading to unnecessary anti-VEGF treatment [8]. In such a report, the spectral-domain optical coherence tomography (SD-OCT) revealed cystoid changes in the PMF resembling macular edema. However, late-phase FFA showed no optic nerve head staining or dye leakage in the macula, differing from typical imaging features of macular edema. In the present case, the cystoid changes are larger and accompanied by outer retinal layer atrophy. Additionally, late-phase FFA shows fluorescence pooling in the macula.

The pathogenesis of retinal abnormalities caused by PM is not yet fully understood. One possible explanation for PMF is that the growth of the outer layers of the eye, such as the sclera, choroid, and RPE, is inhibited, while the development of the retinal nerve fiber layer is not, leading to the formation of retinal folds [15]. In our case, beyond the cystic changes observed in the macula, there was concurrent atrophy of the outer retinal layers. The cystic changes in the macular region and outer retinal atrophy observed on OCT, along with the late-phase fluorescence pooling shown on FFA, all suggest RPE dysfunction, although this may not be solely attributable to PM. Other degenerative macular conditions such as AMD or chronic central serous chorioretinopathy (CSCR) in a high hyperopic eye should be considered. The primary hypothesis for this manifestation is that retinal and choroidal crowding, coupled with scleral thickening, leads to impaired retinal fluid drainage. Prolonged impairment in retinal fluid drainage subsequently results in the decompensation of the outer retinal barrier, specifically the RPE layer. This explains the late-stage clinical and imaging features associated with this disease, such as the characteristic “radiating” low-fluorescence pattern observed on ICGA. Once RPE decompensation occurs, interstitial retinal fluid can no longer be effectively drained, resulting in fluid accumulation (as seen on FFA) and cystic changes in the macula (as observed on OCT). Consequently, anti-VEGF treatment is ineffective in such cases. Abroug et al. [5] describe an abnormal OCT feature of subretinal findings that might correspond to a fibrinous or proteinaceous subretinal material from the choroidal vasculature through marked breakdown of the outer blood–retinal barrier at the RPE level. Our case adds to recently reported cases linking PM and RPE dysfunction.

However, this hypothetical mechanism has limitations. In this case, no peripheral signs of uveal effusion, such as peripheral choroidal detachment, exudative retinal detachment, or leopard-spot fundus, were observed on clinical examination or wide-field imaging. Thus, although the hypothesis regarding impaired fluid drainage and RPE decompensation is logically plausible, the lack of peripheral signs in this case weakens this assumption. Additionally, the RPE dysfunction observed in this case may not be solely attributed to PM. Other etiologies such as AMD, macular dystrophy, or chronic CSCR in a highly hyperopic eye should also be considered. However, the patient had no relevant medical history of the abovementioned diseases; we still consider PM to be more likely.

Since macular edema and atrophy were the primary cause of visual impairment, no reduction in intraretinal fluid or improvement in visual acuity was observed following anti-VEGF therapy.

In conclusion, this case report highlights the importance of understanding the key features associated with the RPE dysfunction caused by PM. Anti-VEGF treatment is not necessary in such conditions.

## Data Availability

The data that support the findings of this study are available from the corresponding authors upon reasonable request.

## Nomenclature

PM: posterior microphthalmos
PMF: papillomacular fold
RPE: retinal pigment epithelium
VEGF: vascular endothelial growth factor
OCT: optical coherence tomography
